# Recovery of plant communities after ecological restoration of forestry‐drained peatlands

**DOI:** 10.1002/ece3.3243

**Published:** 2017-08-29

**Authors:** Tuomas Haapalehto, Riikka Juutinen, Santtu Kareksela, Markku Kuitunen, Teemu Tahvanainen, Hilja Vuori, Janne S. Kotiaho

**Affiliations:** ^1^ Metsähallitus Parks & Wildlife Finland Jyväskylä Finland; ^2^ Metsähallitus Parks & Wildlife Finland Rovaniemi Finland; ^3^ Department of Biological and Environmental Science University of Jyväskylä Jyväskylä Finland; ^4^ Department of Environmental and Biological sciences University of Eastern Finland Joensuu Finland

**Keywords:** anthropogenic disturbance, community composition, extinction debt, immigration credit, mire, recovery debt, resilience, trajectory, vegetation

## Abstract

Ecological restoration is expected to reverse the loss of biodiversity and ecosystem services. Due to the low number of well‐replicated field studies, the extent to which restoration recovers plant communities, and the factors underlying possible shortcomings, are not well understood even in medium term. We compared the plant community composition of 38 sites comprising pristine, forestry‐drained, and 5 or 10 years ago restored peatlands in southern Finland, with special interest in understanding spatial variation within studied sites, as well as the development of the numbers and the abundances of target species. Our results indicated a recovery of community composition 5–10 years after restoration, but there was significant heterogeneity in recovery. Plant communities farthest away from ditches were very similar to their pristine reference already 10 years after restoration. In contrast, communities in the ditches were as far from the target as the drained communities. The recovery appears to be characterized by a decline in the number and abundance of species typical to degraded conditions, and increase in the abundance of characteristic peatland species. However, we found no increase above the drained state in the number of characteristic peatland species. Our results suggest that there is a risk of drawing premature conclusions on the efficiency of ecological restoration with the current practice of short‐term monitoring. Our results also illustrate fine‐scale within‐site spatial variability in the degradation and recovery of the plant communities that should be considered when evaluating the success of restoration. Overall, we find the heterogeneous outcome of restoration observed here promising. However, low recovery in the number of characteristic species demonstrates the importance of prioritizing restoration sites, and addressing the uncertainty of recovery when setting restoration targets. It appears that it is easier to eradicate unwanted species than regain characteristic species by restoration.

## 1 INTRODUCTION

Land‐use changes and degradation of ecosystems by humans are the biggest global threats to biodiversity and ecosystem services (Foley et al., 2005; Hooper et al., 2012; Millennium Ecosystem Assessment 2005). The goal of ecological restoration is the recovery of ecosystems’ original structures and functions (Dobson, Bradshaw, & Baker, 1997; McDonald, Gann, Jonson, & Dixon, 2016; Shackelford et al., 2013; Society for Ecological Restoration International 2004). Consequently, restoration has been highlighted as a major strategy in reversing biodiversity losses and in increasing the provision of ecosystem services (Bullock, Aronson, Newton, Pywell, & Rey‐Benayas, 2011; Wortley, Hero, & Howes, 2013) with an increasing global emphasis in the future (Convention on Biological Diversity 2016). Predicting if restoration goals are likely to be achieved depends on understanding the postrestoration succession of community composition over time in relation to target and degraded communities, as well as the mechanisms affecting the succession (Matthews, Spyreas, & Endress, 2009; Moreno‐Mateos, Meli, Vara‐Rodríguez, & Aronson, 2015; Suding & Hobbs, 2009). Despite a growing number of studies, understanding the effectiveness of ecological restoration requires more studies; due to the lack of replicates or appropriate reference sites, or a timescale of a few years only, surprisingly few studies have provided generalizable results even in medium term (Andersen et al., 2017; Larkin, Bruland, & Zedler, 2016; Moreno‐Mateos et al., 2015).

Instead of increasing the number of species per se, restoration aims most often at regaining original communities. To be more precise, restoration should recover the number and abundance of species characteristic to the original ecosystem and decrease the number and abundance of species characteristic to the degraded ecosystem. Meta‐analyses with large but often heterogeneous data sets highlight the importance of robust restoration methods relying on modification of physical or abiotic environment, such as hydrology, in contrast to often costly biological management interventions (Moreno‐Mateos, Power, Comin, & Yockteng, 2012; Moreno‐Mateos et al., 2015). At the same time, valuable field studies spanning for the first few years after restoration suggest that, because target species were missing after restoration, the modification of physical environment should be supplemented by finer‐scale biological management interventions such as species transplantation (e.g., Hedberg et al., 2012). In pursuit of the most cost‐efficient restoration interventions, it appears very important to understand how efficient the restoration measures without biological management interventions actually are in regaining original species, and, on the other hand, in eliminating the species that have immigrated to the focal ecosystem due to altered environmental conditions. The importance of understanding the potential and timescale of restoration to balance ecosystem degradation and extinctions has also been highlighted from a more theoretical perspective recently (Cronk, 2016; Moreno‐Mateos et al., 2017). In contrast to the often delayed extinctions of species caused by habitat degradation, that is extinction debt (Hanski, 2000; Kuussaari et al., 2009; Tilman, May, Lehman, & Nowak, 1994), restoration should maximize the species or immigration credit, that is, bring back as many original species as possible (Cronk, 2016; Jackson & Sax, 2010).

Boreal and subarctic peatlands cover only 3% of the global land area but store one‐third of global soil carbon (Yu, 2011). Simultaneously, peatlands impact the global climate by emitting CH_4_ and N_2_O (Gong et al., 2013; Juszczak & Augustin, 2013). These important ecosystem functions, which are closely related to the characteristics of peatland plant communities (Ward et al., 2013), are globally threatened (Lappalainen, 1996; Strack, 2008; Tanneberger & Wichtmann, 2011). A major cause of degradation is drainage for timber production affecting approximately 15 million hectares of peatlands (Strack, 2008). Restoration methods of peatlands drained for forestry comprise mainly of recovering the original hydrology by blocking the water flow along the ditches without applying biological interventions such as species transplantations (Andersen et al., 2017; Similä, Aapala, & Penttinen, 2014; Vasander et al., 2003). Although some promising examples on the recovery of peatland vegetation exist from studies lasting for the first few years after restoration (Haapalehto, Vasander, Jauhiainen, Tahvanainen, & Kotiaho, 2011; Hedberg et al., 2012; Laine et al., 2011; Mälson, Backeus, & Rydin, 2008), there are concerns on the effectiveness of restoration in recovering the targeted plant community composition in longer term (Moreno‐Mateos et al., 2012).

The impact of drainage and restoration varies significantly within sites. Drainage alters hydrology by lowering water table level and redirecting water flow. Water table is not affected uniformly over the drained areas but, instead, ditches cause an unnatural systematic pattern where water table is inclined toward ditches (Haapalehto, Kotiaho, Matilainen, & Tahvanainen, 2014; Price, Heathwaite, & Baird, 2003). In addition to the water table pattern, other impacts close to ditches, such as compression by heavy excavators and piling of excavated peat during drainage alongside ditches, contribute to the spatial pattern in the environmental factors within sites. Furthermore, filling of ditches using heavy machinery creates strongly modified and disturbed areas, while areas between ditches may have close‐to‐natural conditions already before the actions. In general, better understanding on such spatial heterogeneity in relation to restoration trajectories is called for (Larkin, 2016; Larkin et al., 2016). Within boreal peatlands, there are indications on spatially related community changes as forestry‐drained peatlands tend to have increased similarity in the landscape due to increase in common forest species postdrainage (Laine, Vasander, & Laiho, 1995). On the other hand, increased within‐site heterogeneity in ecosystem functioning with respect to the ditch line has been reported due to drainage (Minkkinen & Laine, 2006), challenging us to investigate how this spatial heterogeneity interacts with restoration efforts.

Here, we report the effects of drainage and restoration on plant community composition in boreal peatlands from a sample of 38 independent sites located in pristine, drained, and 5 years ago or 10 years ago restored peatlands. More specifically, we asked (1) how plant community composition is changed by drainage lasting for several decades, (2) to what extent plant community composition recovers 5 or 10 years after restoration, (3) how community change and recovery depend on the distance from the drainage ditch, and (4) what is the effect of drainage and restoration on the number and abundance of characteristic peatland species, and on the number and abundance of the unwanted species typical to drained conditions?

## 2 MATERIALS AND METHODS

We selected altogether 38 study sites in southern Finland between 61°53′ and 62°51′N and 22°53′ and 25°26′E. Each site belongs to one of four management categories: (1) pristine (*n *= 10), (2) drained (*n* = 9), (3) previously drained and restored 3–7 years before the study (restored 5 years ago, *n* = 9), (4) previously drained and restored 9–12 years before the study (restored 10 years ago, *n* = 10). From here on, the management categories are referred to as pristine, drained, res 5 and res 10, respectively.

Study sites were selected based on examination of old and new aerial photographs combined with extensive field work to ensure that the drained and restored sites had originally represented the similar vegetation types as the pristine ones (weakly minerotrophic *Sphagnum* dominated pine fens, that is, mainly Oligotrophic ordinary low sedge (pine) fens sensu Eurola et al., 2015). In phytosociological terms, the sites belong to the order Caricetalia fuscae and mainly to the alliance Sphagno‐Caricion canescentis (Mucina et al., 2016). The drained and restored sites were drained for forestry during 1960s and 1970s with ditch interval of 30–50 m. It is likely that as a standard forest management procedure, all the drained and restored sites have been fertilized with mineral fertilizers containing phosphorus and potassium. Restoration measures included filling the ditches with peat excavated near the ditches and partial removal of the tree stands if drainage had markedly increased the growth of *Pinus sylvestris* and *Betula pubescens*. The ditches of the drained and, for convenience, also the infilled ditches of the restored sites are referred to as “ditches” from here on.

A grid of twenty 1‐m^2^ plots was established at each study site (Appendix S1, Fig. S1). At the drained and restored sites, the plots were placed in five parallel transects four meters apart running perpendicular to the ditch. The plots were placed at 0, 5, 10, and 15 meters from the ditch within each transect. At each pristine site, a similar grid of plots was laid. As there were no ditches at the pristine sites, the orientation of the transects was randomized making the pristine sites a good control for the within‐peatland variability and ensuring the robustness of our statistical testing. In an earlier study, we found that drainage had lowered the average water table level during the growing season by 65, 25, 15, or 10 cm, on average, at the distances 0, 5, 10, or 15 m from the ditch, respectively (Haapalehto et al., 2014). Five years after restoration, the average water table level did not differ from that of the pristine sites anymore, and the within‐site variability in the water table level had nearly disappeared (Haapalehto et al., 2014). For more details about the sites, their selection and the study set‐up see Appendix S1.

At each plot, the abundance (percentage cover) of each species of vascular plants and bryophytes (Bryophyta, Marchantiophyta) as well as some species of lichens was estimated to the nearest 1%. Plant community composition data were collected between June and August 2007 by the same two people at each site.

To determine the overall change in plant communities, we calculated dissimilarities of plant communities between all studied vegetation plots using the Bray–Curtis dissimilarity measure. To determine the effect of drainage on plant community composition, we calculated the dissimilarity of each drained 1‐m^2^ plot to all pristine plots. We then calculated the average dissimilarity to pristine plots for each drained plot. Similarly, to determine the effect of restoration, all restored plots were compared to pristine ones, and mean dissimilarity per plot was used. Dissimilarities among pristine plots were calculated similarly as a control to show the extent of natural variation within pristine communities. The dissimilarity of each plot thus expresses the dissimilarity of the plant community composition of the focal plot to the average plant community composition of the pristine reference plots.

The effect of management category (pristine, drained, res 5, res 10), distance (0, 5, 10, or 15 m), and their interaction on the dissimilarity was analyzed with linear mixed models addressing the nested structure of our setup. Fisher's least significant difference (LSD) tests were used for pairwise comparisons to study general differences in dissimilarity between management categories and separately at different distances.

A nonmetric multidimensional scaling (NMS) ordination was performed to further illustrate the interaction between management category and distance, and to examine the trajectories of recovery, that is, the succession of community composition in relation to target communities. Species weighted average scores and classification of species according to their characteristic habitat type (as defined by ecological literature) were used for further interpretation of NMS results.

To investigate the effect of restoration on the number and abundance of characteristic peatland species and unwanted species, we conducted an indicator species analysis (ISA) with the data of drained and pristine sites to identify species especially characteristic to pristine or drained conditions. Thereafter, the total abundance and the number of species indicative of either pristine (characteristic species) or drained conditions (unwanted species) per plot were calculated. The effect of restoration on the number and abundance of characteristic and unwanted species was tested with a linear mixed model analysis. For specific details on the statistical analyses see Appendix S1.

## 3 RESULTS

**Figure 1:**
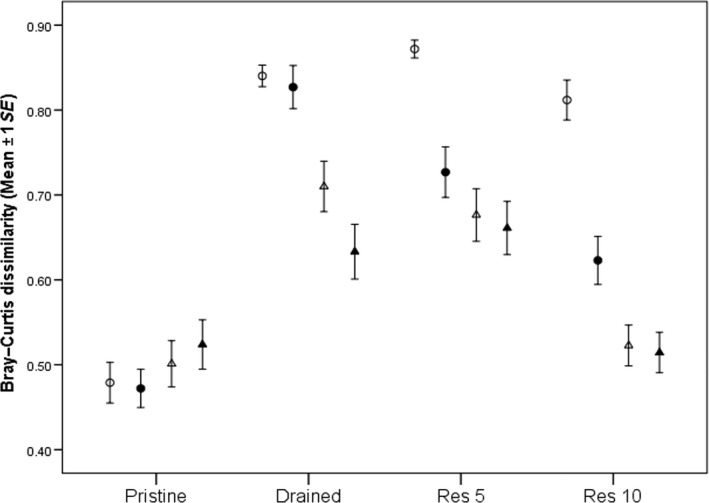
The average Bray–Curtis dissimilarity of plant communities at different distances from the ditch. The lower the dissimilarity, the more similar to pristine the communities are. Open circles, filled circles, open triangles, and filled triangles represent 0, 5, 10, and 15 m distance, respectively. Res 5, restored 5 years ago; Res 10, restored 10 years ago

**Figure 2:**
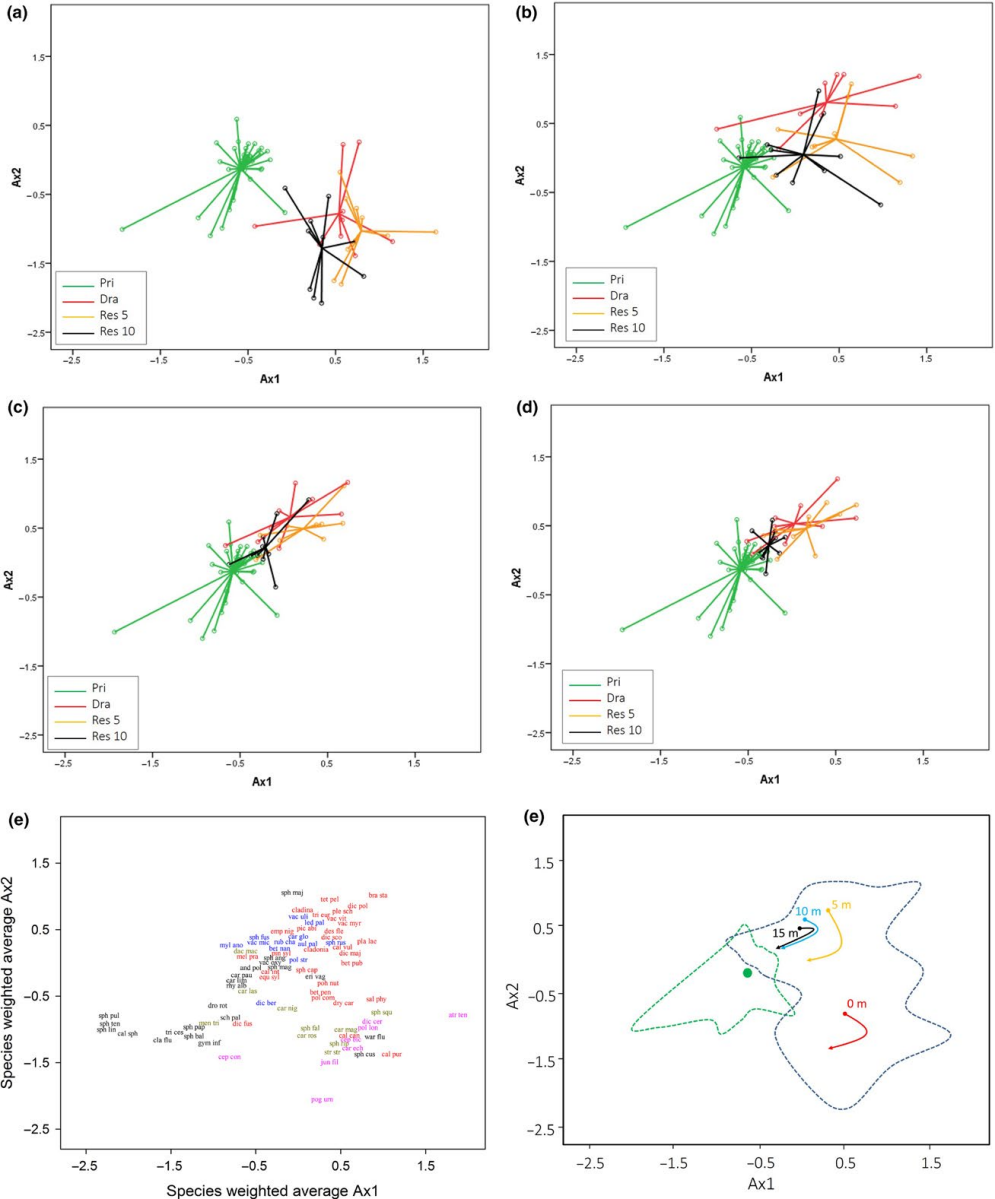
Nonmetric multidimensional scaling (NMS) ordination of vegetation plots of different management categories: green, red, orange, and black circles in panels a–d indicate pristine, drained, 5 years ago restored, and 10 years ago restored, respectively. Different distances from ditch are shown in separate panels (a, b, c, and d for 0, 5, 10, and 15 m, respectively) for drained and restored sites, while the full set of pristine is shown in all panels. The species are divided in groups according to their ecology: black = wet poor peatlands, yellow = wet minerotrophic peatlands, blue = dry poor peatlands (hummocks), red = forests, purple = disturbed surfaces with bare peat and flowing water (panel e). General trajectories of recovery of plant communities (panel f) are derived from group centroids and shown in the same NMS ordination space, where the scatter of pristine and of all other management categories are illustrated with green and dark blue dotted lines, respectively. Green dot represents the centroid of pristine communities, that is, restoration target

Management category and distance had a significant main effect on dissimilarities (i.e., average dissimilarity from pristine plots), and there was an interaction between the two (Table 1, Fig. 1). The dissimilarities of the plant communities of drained sites to pristine were overall greater than the dissimilarities among the pristine peatland plant communities (Table 2, Fig. 1). Consequently, even though there was considerable variation among the pristine peatland communities (Fig. 1), drainage had resulted in a significant overall change in plant community composition. The difference in the dissimilarities depended, however, on the distance from the ditch such that the change in community composition decreased with increasing distance (Table 2, Fig. 1). As compared to the pristine, the communities at the drained sites were characterized by high abundance of forest species and low abundance of species typical to bogs and fens (Fig. 2; Table S1). However, certain species typical to wet fens and bare peat were found in ditches of drained sites in low numbers (Fig. 2; Table S1).

**Table 1:** The fixed effects of the linear mixed model analysis for dissimilarity of plant community composition with management categories (MC; pristine, drained, restored 5 years ago, restored 10 years ago), distance from ditch (0, 5, 10, and 15 m) and their interaction

Source	Denom. *df*	*F*	*p*
Intercept	34.0	1412.05	<.001
MC	34.0	12.10	<.001
Distance	102.0	17.19	<.001
MC*Distance	102.0	4.18	<.001

Numerator *df*'s are 1, 3, 3, and 9 for Intercept, MC, Distance, and MC*Distance, respectively.

**Table 2:** Mean difference (MD) and probability (*p*) of pairwise LSD comparisons for dissimilarity of plant community composition between management categories (MC) overall, and between management categories at different distances from ditch

MC(I)	MC(J)	Overall		0 m		5 m		10 m		15 m	
		MD (I–J)	*p*	MD (I–J)	*p*	MD (I–J)	*p*	MD (I–J)	*p*	MD (I–J)	*p*
Pri	Dra	**−0.259**	**<.001**	**−0.361**	**<.001**	**−0.355**	**<.001**	**−0.209**	**.002**	**−**0.109	.102
	Res5	**−0.240**	**<.001**	**−0.394**	**<.001**	**−0.255**	**<.001**	**−0.175**	**.009**	**−0.137**	**.041**
	Res10	**−0.124**	**.014**	**−0.333**	**<.001**	**−0.151**	**.021**	**−**0.022	.738	0.009	.883
Dra	Res5	0.018	.717	−0.033	.632	0.100	.143	0.034	.622	−0.028	.683
	Res10	**0.135**	**.009**	0.028	.669	**0.204**	**.003**	**0.187**	**.006**	0.119	.076
Res5	Res10	**0.116**	**.023**	0.061	.359	0.104	.120	**0.154**	**.022**	**0.147**	**.029**

MC, management categories; Pri, pristine; Dra, drained; Res5, restored 5 years ago; Res10, restored 10 years ago.

Statistically significant values (p<.05) are shown in bold.

Dissimilarities of the res 5 communities did not differ from those of the drained communities overall, or at any distance (Table 2, Fig. 2). At the res 10 sites, the dissimilarities were overall reduced relative to those of drained sites (Table 2). While there were no changes at 0 m, the reductions in the dissimilarities at other distances were evident (Table 2; Fig. 2). Overall, dissimilarities of the res 5 and res 10 communities were greater than those of the pristine, but the differences were dependent on the distance (Table 2, Fig. 2). The plots at the res 5 peatlands had greater dissimilarities than the pristine sites at all distances, but the dissimilarities decreased with increasing distance (Table 2). The plots at the res 10 sites showed a similar pattern, but importantly, there was no significant difference between res 10 and the pristine at 10‐ or 15‐m distance from the ditch (Table 2). As compared to the drained peatlands, both of the restored peatland communities were characterized by higher abundance of peatland species with wide ecological niches (e.g., *Sphagnum angustifolium* and *Sphagnum magellanicum*; Fig. 2e, Table S1) and less influenced by forest species at distances from 5 to 15 m. In contrast, the relative importance of species capable of utilizing the bare peat surfaces and released nutrients (e.g., *Carex magellanica*,* Eriophorum vaginatum*; Silvan, Tuittila, Vasander, & Laine, 2004) seemed to increase 5 years after restoration at 0 m distance (Table S1). This was followed by a decreased relative importance of species typical to bare peat and increased relative importance of species typical to natural wet fens 10 years after restoration (Fig. 2; Table S1).

When we explored the trajectories of community composition succession at different distances of sites belonging to different management categories, a roughly similar pattern of succession was found. After initially increased abundance of species typical to heavily degraded habitats (right to bottom‐right), all of the trajectories change track more or less toward the pristine sites (bottom‐left, Fig. 2f). In contrast to 5–15 m plots, the trajectory of communities at 0 m distance does not point toward communities of the pristine sites.

**Figure 3:**
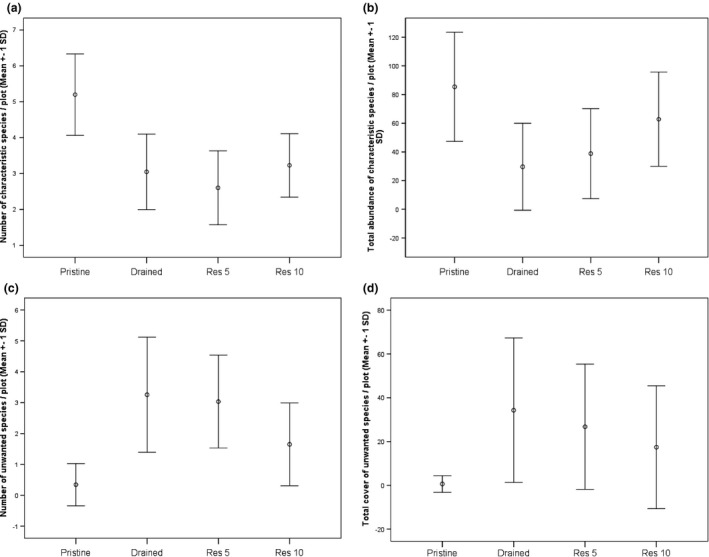
The mean (±1 *SD*) number (a) and abundance (b) of characteristic peatland species (species indicative of pristine) and the number (c) and abundance (d) of unwanted species (species indicative of drained) per vegetation plot

According to ISA, there were eight species indicative of pristine and 13 species indicative of drained peatlands (characteristic and unwanted species, respectively; Table 3). There was a difference in the number and abundance of characteristic species and unwanted species per plot between management categories (Table 4). The number of characteristic species was lower in res 5 and res 10 than in pristine, and it did not differ significantly from drained in either of the cases (Table 5, Fig. 3). The average number of characteristic species was, however, higher in res 10 than in res 5 sites (Table 5, Fig. 3). The total abundance of characteristic species per plot was lower in both res 5 and res 10 than in pristine peatlands (Table 5, Fig 3). However, while there was no significant difference between drained and res 5, the abundance of characteristic species was higher in res 10 than in drained or res 5 (Table 5, Fig. 3). The number of unwanted species was significantly higher in res 5 and res 10 than in pristine (Table 5, Fig 3). While there was no significant difference between drained and res 5, the number of unwanted species was lower in res 10 than in drained and res 5 (Table 5, Fig. 3). The abundance of unwanted species was on average lower in pristine than in res 5 and res 10 (Table 5, Fig. 3). There was no significant difference in total abundances between drained and res 5, but the abundance was on average lower in res 10 than in drained (Table 5, Fig. 3).

**Table 3:** Summary of indicator species analysis (ISA) of plant species. Only the species with a significant observed maximum indicator values of pristine or drained peatlands of Monte Carlo test are shown

	Observed indicator value	Indicator value from randomized groups		
		Mean	*SD*	*p*
Species indicative of pristine plots				
* Andromeda polifolia*	94.8	63.6	9.48	<.001
* Carex pauciflora*	95.2	46.8	10.08	<.001
* Carex rostrata*	73.4	47.2	10.44	.020
* Drosera rotundifolia*	93.8	50.5	12.81	.002
* Eriophorum vaginatum*	64.4	54.8	3.64	.012
* Sphagnum angustifolium*	74.1	55.9	4.42	<.001
* Straminergon stramineum*	54.7	32.6	9.71	.040
* Vaccinium oxycoccos*	76.6	57.1	5.32	.001
Species indicative of drained				
* Betula pubescens*	99.7	40.0	11.38	<.001
* Calluna vulgaris*	44.4	23.1	9.18	.035
* Carex canescens*	88.4	37.9	11.21	<.001
* Cladonia sp*.	79.0	40.7	11.78	.004
* Dicranella cerviculata*	66.7	27.6	9.92	.004
* Dicranum polysetum*	87.5	37.6	11.46	<.001
* Picea abies*	76.9	36.1	10.58	.003
* Pleurozium schreberi*	99.8	56.3	11.12	<.001
* Polytrichastrum longisetum*	55.6	25.1	10.57	.013
* Sphagnum riparium*	55.7	31.2	10.66	.037
* Sphagnum russowii*	95.2	75.7	9.01	.006
* Vaccinium myrtillus*	77.6	37.5	11.55	.002
* Vaccinium vitis‐idaea*	82.8	45.3	12.55	.005

**Table 4:** The fixed effects of the linear mixed model analysis for the number and abundance of original characteristic peatland species and unwanted species with management category (MC; pristine, drained, restored 5 years ago, restored 10 years ago)

Source	Characteristic species				Unwanted species			
	Number		Abundance		Number		Abundance	
	*F*	*p*	*F*	*p*	*F*	*p*	*F*	*p*
Intercept^a^	254.31	<.001	1,470.03	<.001	303.97	<.001	70.65	<.001
MC^b^	13.710	<.001	40.173	<.001	32.670	<.001	9.613	<.001

a Numerator and denominator *df*s are 1 and 34, respectively.

b Numerator and denominator *df*s are 3 and 34, respectively.

**Table 5:** The pairwise LSD comparisons for the number and abundance of characteristic peatland species and unwanted species between management categories (MC)

		Characteristic species				Unwanted species			
		Number		Abundance		Number		Abundance	
		MD (I–J)	*p*	MD (I–J)	*p*	MD (I–J)	*p*	MD (I–J)	*p*
MC (I) MC (J)									
Pri	Dra	**2.15**	**<.001**	**55.77**	**<.001**	**−2.91**	**<.001**	**−33.67**	**<.001**
	Res 5	**2.59**	**<.001**	**46.58**	**<.000**	**−2.69**	**<.001**	**−26.14**	**<.001**
	Res 10	**1.97**	**<.001**	**22.63**	**.021**	**−1.31**	**<.001**	**−16.80**	**.014**
Dra	Res 5	0.44	.105	−9.19	.358	0.22	.523	7.53	.278
	Res 10	−0.18	.493	**−33.14**	**.002**	**1.61**	**<.001**	**16.87**	**.016**
Res 5	Res 10	**−0.62**	**.022**	**−23.95**	**.018**	**1.38**	**<.001**	9.34	.169

Pri, pristine; Dra, drained; Res 5, restored 5 years ago; Res 10, restored 10 years ago.

Statistically significant values (p<.05) are shown in bold.

## 4 DISCUSSION

Positive changes in certain species groups such as wetland bryophytes and sedges have been observed in a few years after peatland restoration (Haapalehto et al., 2011; Hedberg et al., 2012; Laine et al., 2011; Maanavilja, Aapala, Haapalehto, Kotiaho, & Tuittila, 2014; Mälson et al., 2008). A relatively rapid increase toward the original abundance for species such as *S. angustifolium* and *Sphagnum russowii* can also be seen in our data during the first 5 years after restoration (Table S1). Examination of the succession of entire community composition suggests, however, that despite the rapid recovery of certain species, restoration actually pushes the community composition initially in a direction totally different from the target community composition. Significant succession toward target communities can be seen only about 10 years after restoration. Furthermore, we found that different components of community composition reacted differently to the restoration. While there was a significant overall change in community composition toward the pristine target, the amount of characteristic species was at the level of the drained sites still 10 years after restoration. In addition, community changes were dependent on the location of the study plots within the sites, that is, on the distance from the ditch. Together our findings suggest that with the common tendency toward low‐intensity monitoring schemes that typically last only for the first few postrestoration years (Moreno‐Mateos et al., 2012, 2015), there is a risk of drawing premature and unjustified conclusions about the effectiveness of restoration, especially if the schemes rely heavily on plant communities. The importance of this issue is highlighted by the fact the European Union LIFE funding invested 167.6M € in peatland restoration between 1993 and 2015 alone with often insufficiently planned monitoring schemes that typically lasted only a few years (Andersen et al., 2017).

There are concerns that degradation of ecosystems decrease their spatial heterogeneity and, consequently, increase the homogeneity of species communities and habitats (Larkin, 2016). However, man‐made disturbances rarely impact an ecosystem uniformly, especially in case of drainage, where very heterogeneous hydrological conditions and variable shading by the tree canopy are created within the site (Haapalehto et al., 2014; Laine et al., 1995). It was therefore expected that we would find more altered communities within and close to the ditches than at 10–15 distance. In fact, the drained ecosystem undergoes another disturbance with spatially heterogeneous impact due to restoration. The areas within and close to the ditches are heavily impacted by a significant raise in the water table as well as by excavation and piling of peat for damming, whereas the areas further away from the ditches are mainly impacted by a smaller rise of water table level (Haapalehto et al., 2014).

While spatial heterogeneity is often encouraged to enhance species establishment and provision of ecosystem services, better understanding on the relationship between recovery trajectories and spatial heterogeneity is needed (Larkin et al., 2016). Our results suggest that very different trajectories may exist within a restored site due to the heterogeneous impacts of previous land‐use and restoration measures themselves. Firstly, there was variability in how similar to the target the communities developed within a decade after restoration. While the communities of the 10 years ago restored sites were already very similar to the pristine composition at 10–15 m distance, the communities within the filled ditches were still as dissimilar to the pristine as the communities in the ditches of the drained sites. Secondly, there appeared to be systematic heterogeneity due to drainage and restoration in how much the communities change after restoration. The mean differences in the dissimilarities at different distances between drained and res 10 suggest that the change toward the target was greatest in the 5 m distance from the ditch (see Table 2, MD for both measures), apparently mainly due to largest changes in water table level after restoration within site (Haapalehto et al., 2014). In contrast, community changes appeared to be rather subtle at 10–15 m distance. The comparison of mean differences and trajectories of community succession suggests, furthermore, that significant changes take place in communities within the ditches (Table 2, Fig. 2e). The development of community composition was, however, not toward the target, and the trajectory of community succession within filled ditches still pointed away from the target 10 years after the restoration.

Importance of understanding the potential and timescale of restoration to balance ecosystem degradation and extinctions has been highlighted recently (Cronk, 2016; Moreno‐Mateos et al., 2017). In contrast to the often delayed extinctions of species caused by habitat degradation, that is extinction debt (Hanski, 2000; Kuussaari et al., 2009; Tilman et al., 1994), restoration should maximize the species or immigration credit, that is, bring back as many original species as possible (Cronk, 2016; Jackson & Sax, 2010). The species/immigration credit should, consequently, balance the species eradications caused by ecosystem degradation (Jackson & Sax, 2010). Extinction debt and species credit can be interpreted from changes in the pool of characteristic species (Helm, Hanski, & Pärtel, 2006), such as the results of indicator species analysis here. It is therefore fruitful to look at our results from this more theoretical perspective. We found a lower number of characteristic peatland species from drained than from pristine sites suggesting that drainage has led to eradication of characteristic peatland species in a time span of ca. 50 years. The number of characteristic peatland species did not differ between the drained and res 10 sites, and it was higher for pristine than for either drained or res 10. Consequently, in terms of changes in the pool of characteristic species, restoration appeared not to be able to recover the number of species eradicated by drainage within the studied 10‐year period. Considering changes in the pool of unwanted species, we found a lower number of species at res 10 than drained sites. Hence, it appears that restoration was successful in eradicating unwanted species within the same period. Taken together the changes in the pools of characteristic peatland species and unwanted species, it appears that it was easier to eradicate unwanted species than to bring back characteristic peatland species within the studied postrestoration period.

Strength of degradation in environmental quality is one of the main factors affecting the time lag of extinctions (Kuussaari et al., 2009). Here, the rise of the water table after the restoration (Haapalehto et al., 2014) seemed effective in decreasing the habitat quality for unwanted species to the extent that resulted in their eradication within the comparably rapid timescale. On the other hand, the immigration of characteristic species is expected to be delayed to some extent as the re‐establishment of a eradicated populations requires several subsequent successes: dispersal of propagules, establishment of individuals, and survival to reproductive maturity and persistence of populations via continued reproduction (Jackson & Sax, 2010). However, the relatively poor recovery in the pool of characteristic species raises the question, will restoration without active transplantations bring back the disappeared species, or will we end up with less diverse communities than originally? Indeed, a significant recovery debt may remain after restoration, and the recovery of plant communities may remain imperfect even several decades after restoration (Moreno‐Mateos et al., 2012, 2015, 2017). Species with narrow ecological niches and poor dispersal abilities, such as *Scheuchzeria palustris* and *Carex limosa* here (see Table S1), are among species that are often reported to be missing still a decade after peatland restoration (e.g., Haapalehto et al., 2011; Hedberg et al., 2012). On the other hand, our extensive spatial sampling reveals that certain characteristic peatland species (e.g., *Carex rostrata* and *Straminergon stramineum*) grow almost exclusively in the pits, pools, and wettest surfaces of the filled ditches, that is, the most heavily disturbed areas in our case. This way the parts of restored peatlands furthest away from the target may actually complement the ecosystem‐level diversity during the early recovery (see Table S1).

Results from the ISA suggest that restoration increased the abundance of characteristic peatland species and decreased the abundance of unwanted species. It appears that especially the characteristic peatland species with relatively wide niches such as *S. angustifolium* and *S. magellanicum* were able to survive in low abundances over the drainage period. They could proliferate clonally from relict populations and colonize new areas rapidly after the recovery of suitable environmental conditions (see Table S1). The fast recovery of the abundances in remnant populations plays an important role in the early recovery of the ecosystem functioning (Kareksela et al., 2015; Maanavilja, Kangas, Mehtätalo, & Tuittila, 2015). On the other hand, the rapid recovery of certain competitive species is suspected to hinder the re‐establishment of other species later on (e.g., Hedberg et al., 2012). We find, however, that such inhibition, for example, by *Sphagnum* species is more likely in ecosystems with more profound turnover in species pool due to degradation, and larger share of poorly competing species that are strictly confined to environmental conditions typical to natural sites (especially high pH, low nutrient availability).

Taken together, we find the heterogeneous outcome of restoring forestry‐drained peatlands without biological interventions promising. Most of the area is developing toward the target communities within a timescale very short for the development of natural‐state peatlands and plant communities, in general (Cronk, 2016). Furthermore, more heavily disturbed and least recovered areas of the heterogeneous sites appear to provide novel environments for some characteristic species. The comparably low recovery in the number of characteristic species highlights, however, the need for prioritization between restoration sites; natural community composition will be more likely regained by targeting restoration to sites where original target species still exist. In addition to demonstrating the need for spatial prioritization, these results have implications, for example, on biodiversity offsetting, which means compensating for environmental damage caused by land‐use changes in one location by restoring ecosystems elsewhere (Laitila, Moilanen, & Pouzols, 2014; Maron et al., 2012). Offsetting schemes and the principle of “no net loss” of biodiversity have been embraced for wetlands and other ecosystems lately by governments, multinational corporations, and financial institutions (e.g., Bull, Gordon, Watson, & Maron, 2016). If compensations aim at regaining the same amount of characteristic species by restoration as is lost by development projects elsewhere, we need a lot more research on how much restoration is enough to compensate certain amount of losses. This is especially noticeable because, for a few reasons, studied boreal forestry‐drained peatlands appear easy to restore as compared to many other wetland types, in general. Firstly, forestry‐drained peatlands are less degraded than several other types of restored habitats; remnant populations of target species, and a relatively good potential to recover original conditions enhance the probability for restoration success in forestry‐drained peatlands as compared to, for example, former peat mining areas or peatlands with long‐term agricultural use (see e.g., Chimner, Cooper, Wurster, & Rochefort, 2016; van Diggelen et al., 2015; Lamers et al., 2015). Secondly, due to comparably low population density, the fragmentation of the landscape, airborne and waterborne pollution, and suppression by alien species are less likely to hamper restoration in the study area and in northern Europe than in the more densely populated areas, for example, in central Europe and northern America. Thirdly, restoration of forestry‐drained peatlands is a one‐off measure intended to allow a spontaneous succession of communities toward the natural target state by first recovering suitable environmental conditions. In cases where the target state is result of previous human land use, such as often, for example, with rich fens, continuous management is needed to maintain the desired state.

## CONFLICT OF INTEREST

Authors have no conflict of interest to declare.

## AUTHORS’ CONTRIBUTIONS

TH and JSK conceived the ideas and designed methodology; SK and RJ collected the data; TH, JSK, TT, and HV analyzed the data; TH led the writing of the manuscript. All authors contributed critically to the drafts of the manuscript and gave final approval for publication. Authors have no conflicts of interest to declare.

## DATA ACCESSIBILITY

Data will be made available via Dryad.
