# Supplementary figures and images for: Recovery of plant communities after ecological restoration of forestry‐drained peatlands
Source: Ecol Evol. 2017 Aug 29;7(19):7848–58. doi: 10.1002/ece3.3243 (PMC5632633; doi:10.1002/ece3.3243)

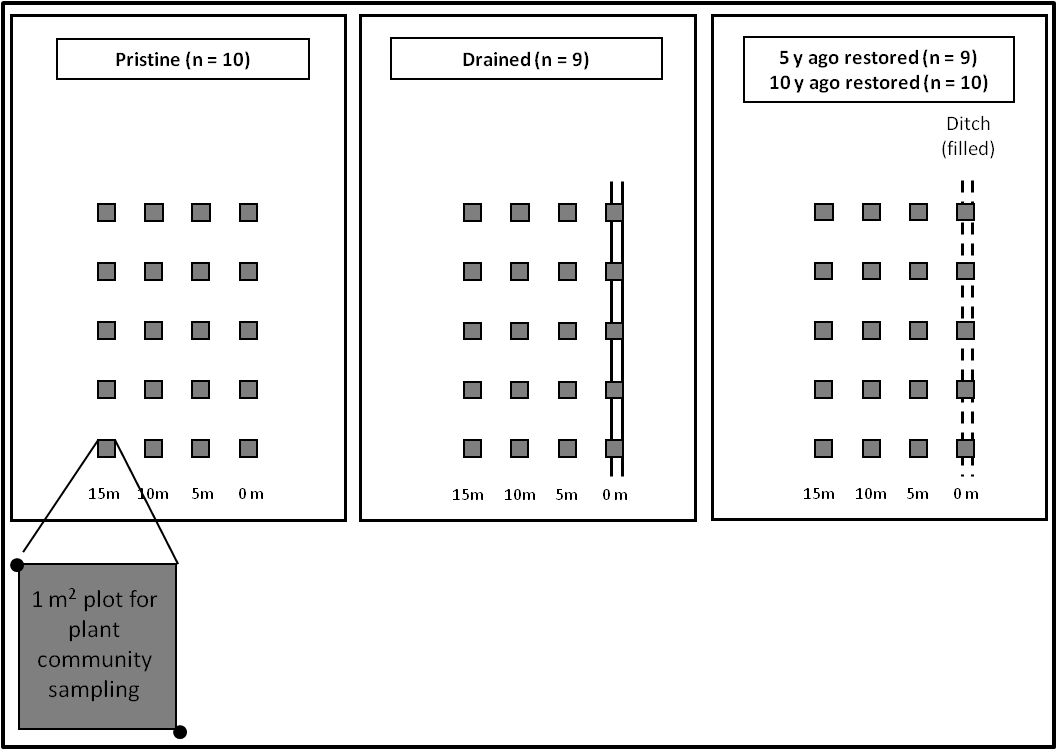

Supplement: Supplementary file 1 [file ECE3-7-7848-s001.tiff]
