# Supplementary material for: Recovery of plant communities after ecological restoration of forestry‐drained peatlands
Source: Ecol Evol. 2017 Aug 29;7(19):7848–58. doi: 10.1002/ece3.3243 (PMC5632633; doi:10.1002/ece3.3243)
Supplement: Supplementary file 2 [file ECE3-7-7848-s002.docx]

**Supplementary Material, Haapalehto et al. 2017, Appendix S1**

**Detailed information on studied sites and their selection**

The 38 study sites are located in the south-boreal climatic-phytogeographical zone, where raised bogs are the main type of greater peatland formations. The sites were located within a radius of 75 km with a range of 200 m - 150 km between the sites. The mean annual temperature is ca. +4°C, precipitation ca. 650 mm and elevation above the sea level ca. 150 meters. The area belongs to the Early Proterozoic bedrock area, characterized by silicaceous granite and granodiorite minerals.

Based on topographic data and field observations, the sites were determined to be independent from each other in their surface water flow. Each site consisted of a hydrologically separable peatland zone of similar vegetation type. The sizes of sites were not recorded precisely but they ranged from a few hundred square meters to a few hectares within each management status category. Average peat depth at the sites ranged from 95 cm to more than 200 cm. The peat was mainly underlain by unsorted glacial till which is typical to the region. In the surroundings of some sites, also esker formations with more sorted sand were found. In 1980s some of the drained sites were designated to conservation with a subsequent decision to restore them. The restoration was conducted by Metsähallitus Parks & Wildlife Finland, which is a state agency responsible for management of conservation areas. During restoration the removal of tree cover was adjusted to roughly mimick the pre-disturbance tree cover in cases where it was seen ecologically necessary by the practitioners. However, due to practical reasons, tree removal had not been possible to full extent in all cases, resulting in slightly larger and more variable stand volumes for res 5 (mean 66.7 m^3^/ha, SD 45.8) and res 10 (mean 43.9 m^3^/ha, SD 37.8) than for pristine sites (mean 8.0 m^3^/ha, SD 5.94). The mean stand volume of drained sites was 64.5 m3/ha. There was also some variation within the stands of the drained sites (SD 61.0) due to thinnings that had taken place at some of the drained sites as a standard forestry measure during the past couple of decades. Decomposition and compression of the peat near the ditches causes subsidence of peat surface and therefore, the filling of the ditches was supplemented by peat dams. The dams elevate ca. 50 cm over the peat surface and extend a few meters at both sides of the ditch to prevent water flow along the subsided area near the ditches. The restoration measures form pits and bare peat surfaces around the filled ditches and thus result in a variable surface structure at the exact site of restoration, while peatland surfaces outside the ditches remain largely intact. At some of the Drained sites ditches had been cleared again in 1990s. Sites were considered pristine if they did not have ditches or other structures used for altering hydrology. Although we did not observe signs of any such effects, it is possible that forestry measures elsewhere in the catchments may have slightly affected hydrology of some Pristine sites. Within each of the 38 study sites, the location of the first plot (laid on the ditch at drained and restored sites or on random surface on pristine sites) was randomized when establishing the study set up.

The set up may be considered a chronosequence. Such space-for-time substitutions are sometimes problematic in inferring vegetation succession e.g. if the sites differ originally in their properties (Johnson & Miyanishi 2008). We have responded to this challenge by selecting a number of replicates for each management status. Additionally, we utilized all available data sets i.e. electronic data bases of Metsähallitus, aerial photos from present time and the time before degradation and restoration, topographic maps and expert field work for selection of suitable sites. Changes take place in peatland ecosystems after drainage and restoration, and the possibility for slightly larger variation in the original vegetation types among drained and restored sites, when compared to pristine sites, cannot be ruled out. However, development of vegetation and tree stands after drainage is fairly well known due to substantial literature on peatland forestry (e.g. Laine et al. 1995, Laine & Vasander 1996). This allows a much more reliable determination of original peatland types than e.g. when studying more severely disturbed systems like peat excavation areas or peat fields. Therefore, we expect that possible marginal differences are not likely to provide grounds for false interpretation of the results and misleading conclusions.

**Supplementary information on statistical analysis**

**Similarity indices**

Bray-Curtis dissimilarity *D* based on the species abundance data between plant communities *i* and *h* was calculated as


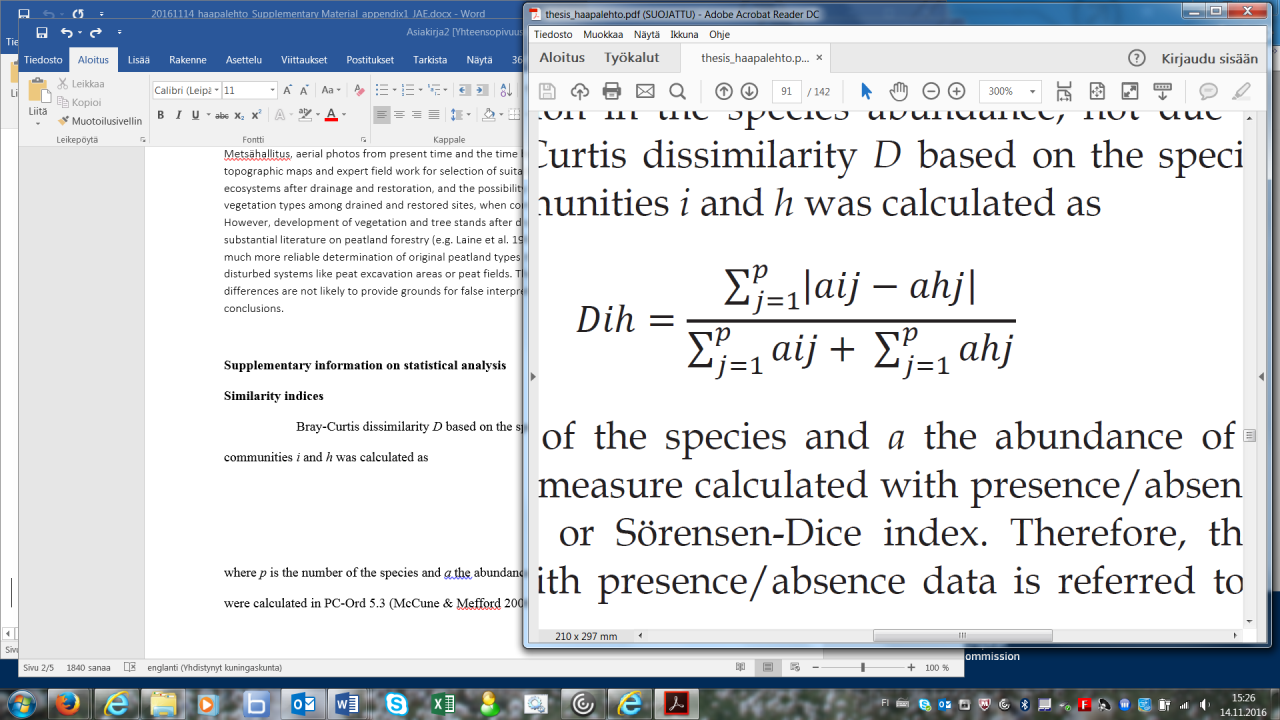


where *p* is the number of the species and *a* the abundance of each of the *j* species. The indices were calculated in PC-Ord 5.3 (McCune & Mefford 2006).

**Linear mixed models**

Management category, distance from the ditch and their interaction were entered as fixed factors in the analysis, and to account for non-independence of data from plots co-occurring within the same site in a hierarchical structurethe study site and distance (subject to study site) as random factors.

All Dissimilarities between the plots within one study site were excluded from the LMM and LSD analyses. Linear mixed models and LSD tests were performed in IBM SPSS Statistics 20.

**NMS**

The differences in community compositions between management categories were inspected by plotting the NMS ordination scores for each management category. This was done separately for each distance from the ditch (Fig. 2 panels a-d). The average location for each management category in the NMS space was, furthermore, depicted as centroids. To relate the plant community composition patterns shown in the Fig. 2 (panels a-d) to the ecology of the observed species, we calculated the weighted average scores for the species in the NMS analyses and plotted them along the NMS axes 1 and 2 (Fig. 2, panel e). To relate the development of community composition to species ecology, we subjectively divided the species in the NMS plot into five categories (wet poor peatlands, wet minerotrophic peatlands, dry poor peatlands (hummocks), forests, disturbed surfaces with bare peat and flowing water) according to their characteristic habitat type (Fig. 2, panel e). To further inspect the trajectories of recovery in relation to distance from the ditch (Fig. 2 panels a-d), we joined with a line the above centroids for each distance (Fig. 2, panel f).

The data on the ecology and characteristic habitats of species was derived from Ulvinen *et al*. 2002 and Eurola *et al.* 1995 for bryophytes and vascular plants, respectively. Species with only one observation were excluded from the ordination analysis. Before the analysis, the data consisting of relative abundances of species within 1-m^2^ plots was relativized by rows (plots) and columns (species) and arcsine square root transformed. The analysis was performed using Bray-Curtis dissimilarity measure, originally six possible axes, 500 iterations in maximum, 250 runs with real and randomized data. As suggested by the analysis software, the 2D solution with a final stress of 21.35 was selected. This resulted in a cumulative r^2^ of 0.75 for the two axes. The NMS ordination was performed with PC-Ord 5.3.

Judged from the ecology of species in the NMS analysis (Fig. 2e), there appear to be two patterns in the NMS ordination data. The first is a gradient running from bottom left to top right corner of the ordination. Species along this gradient are changing from species characteristic to wet and open hollow level habitats in the bottom left end (e.g. *Sphagnum tenellum*, *Sphagnum balticum*, *Cladopodiella fluitans*) through species characteristic to hummock habitats in the middle (e.g. *Sphagnum fuscum*, *Rubus chamaemorus*, *Aulacomnium palustre*) to species characteristic to dry and closed forest habitats in the top right end (e.g. *Vaccinium myrtillus*, *Vaccinium vitis-idaea*, *Pleurozium schreberi*, *Dicranum polysetum*) (Fig. 2e). The other pattern is the clustering and separation of species of highly disturbed bare peat and flowing water habitats to the bottom right corner of the ordination (e.g. *Polytrichastrum longisetum*, *Carex canescens*) (Fig. 2e).

**ISA**

To investigate the effect of restoration on the abundance and numbers of characteristic peatland species and unwanted species, we conducted an Indicator Species Analysis (ISA) with the data of drained and pristine sites only. ISA is a method used for finding indicator species and species assemblages characterizing groups of sites, drained and pristine conditions in this case (Dufrene & Legendre 1997). The average abundance over the vegetation plots for each species at each site was calculated to avoid pseudoreplication in the analysis and the species at p < 0.05 for 4,999 Monte Carlo permutations were considered to be indicators of either pristine or drained conditions. Thereafter, the total abundance (the sum of covers in percent at each plot) and the number of species indicative of either pristine (characteristic species) or drained conditions (unwanted species) were calculated. The effect of drainage and restoration on the number and the abundance of the species indicative of either pristine or drained conditions was thereafter tested with a linear mixed model analysis. The analysis was chosen because it allows the use of data with unequal variances and data from a nested hierarchy like our set-up with altogether 20 vegetation plots at within each site. Management status was used as a fixed factor and as a random factor (subject to site) in the analysis. In addition to the data of Pristine and Drained sites, data from five and 10 years ago restored sites was used for testing. For simplification, all the species determined by ISA to be indicators of Drained peatlands, are referred to as unwanted species. Note, however, that some of these species can be found from other types of pristine peatlands.

**The use of semimetric indices in statistical analyses**

Bray-Curtis is a semimetric index (e.g. Wilson 1931, McArdle & Anderson 2001). This implies that the dissimilarities do not, strictly, fulfil all metric assumptions for testing the differences with ANOVA style linear models (see e.g. McArdle & Anderson 2001). Consequently, non-parametric multivariate analyses relying on permutations for achieving p-values are suggested for testing multivariate data derived from semimetric indices (Legendre & Anderson 1999; Anderson 2001, McArdle & Anderson 2001). Even after deep consideration of possible options we could not find a non-parametric analysis that permutes the data from this type of complex and hierarchical design correctly. Because Bray-Curtis provides a more ecologically meaningful interpretation of the data than metric indices (see McArdle & Anderson 2001), we chose to use Bray-Curtis and linear mixed models as explained above. We expect that our analyses, supported by visual interpretation of Fig. 1 & Fig 2, are not likely to provide grounds for false interpretation of the results and misleading conclusions. This was further verified by running PERMANOVA+ and PERMDISP Anderson *et al.* 2008 with the most suitable analysis parameters available for this kind of data, and comparing the results (not shown here) to the results obtained by linear mixed models.

**References for Appendix S1**

Anderson, M.J. 2001. A new method for non-parametric multivariate analysis of variance. Austral Ecology 26: 32–46

Anderson, M., Gorley, R.N., & Clarke, K.R. 2008. PERMANOVA+ for Primer: Guide to software and statistical methods. Primer-e, Plymouth, UK

Dufrene, M. & Legendre P. 1997. Species assemblages and indicator species: the need for a flexible asymmetrical approach. Ecological Monographs 67(3): 345-366.

Eurola, S., Huttunen, A., & Kukko-Oja, K. 1995. Suokasvillisuusopas. *Oulanka Reports* 14: 1–85

Johnson E.A. & Miyanishi K. 2008. Testing the assumptions of chronosequences in succession. Ecology Letters 11: 419–431

Laine, J., Vasander, H. & Laiho, R. 1995. Long-Term Effects of Water Level Drawdown on the Vegetation of Drained Pine Mires in Southern Finland. Journal of Applied Ecology 32(4):785-802.

Laine, J. & Vasander, H. 1996, Ecology and vegetation gradients of peatlands. In Vasander, H. (ed.) *Peatlands in Finland*, pp. 10-19. Finnish Peatland Society, Helsinki.

Legendre, P., & Anderson, M.J. 1999. Distance-Based Redundancy Analysis: Testing Multispecies Responses in Multifactorial Ecological Experiments. *Ecological Monographs* 69: 1–24.

McArdle, B.H., & Anderson, M.J. 2001. Fitting multivariate models to community data: a comment on distance-based redundancy analysis. *Ecology* 82: 290–297.

McCune, B., & Mefford, M.J. 2006. *PC-ORD. Multivariate Analysis of Ecological Data. Version 5.33.* MjM Software, Gleneden Beach, Oregon, USA.

Ulvinen, T.,Syrjänen, K. & Anttila, S. 2002. Suomen sammalet – levinneisyys, ekologia, uhanalaisuus. 2. Edition. Suomen ympäristö 560. 354 p. The Finnish Environment Institute, Helsinki, Finland.

Wilson, W.A. 1931. On Semi-Metric Spaces. *American Journal of Mathematics* 53: 361–373.

**Appendix S1, Figure S1.**


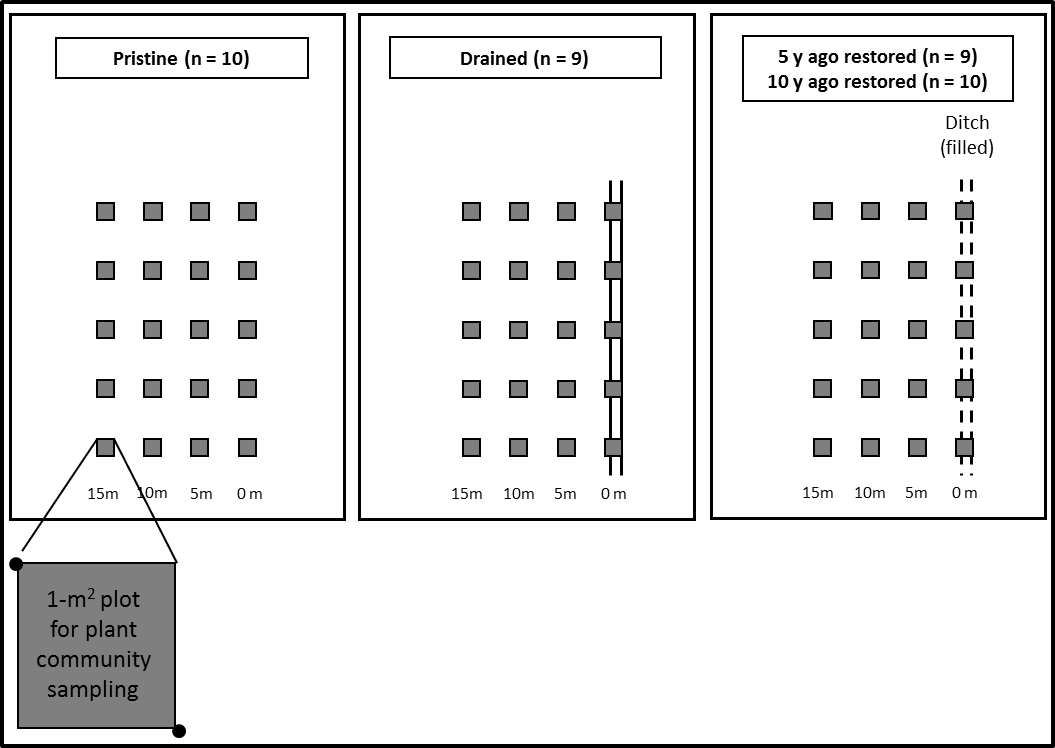


Figure S1. Study set ups for sampling the vegetation at pristine, drained and restored sites. Distances (m) refer to distance of 1-m^2^ plots from ditches at restored and Drained sites. A similar grid of plots was laid at pristine sites.

**Table captions: Supplementary material: Table S1.** Species relative abundance and the number of sites a species has been observed with the management category and distance from the ditch.
